# Eviction filings during bans on enforcement throughout the COVID-19 pandemic: an interrupted time series analysis

**DOI:** 10.17269/s41997-023-00813-1

**Published:** 2023-08-15

**Authors:** Erika M. Brown, Rahim Moineddin, Ayu Hapsari, Peter Gozdyra, Steve Durant, Andrew D. Pinto

**Affiliations:** 1 California Policy Lab, Institute for Research on Labor & Employment, University of California, Berkeley, Berkeley, CA USA; 2 Social Interventions Research & Evaluation Network, University of California, San Francisco, San Francisco, CA USA; 3 Department of Family and Community Medicine, Faculty of Medicine, University of Toronto, Toronto, ON Canada; 4 Upstream Lab, MAP/Centre for Urban Health Solutions, Li Ka Shing Knowledge Institute, Unity Health Toronto, Toronto, ON Canada; 5 Institute for Clinical Evaluative Sciences, Toronto, ON Canada; 6 Department of Family and Community Medicine, St. Michael’s Hospital, Toronto, ON Canada; 7 Dalla Lana School of Public Health, University of Toronto, Toronto, ON Canada

**Keywords:** Housing insecurity, Social determinants of health, Intersectoral collaboration, Health policy, COVID-19 pandemic, Residential evictions, Précarité du logement, déterminants sociaux de la santé, collaboration intersectorielle, politique de santé, pandémie de la COVID-19, expulsions résidentielles

## Abstract

**Objective:**

Bans on evictions were implemented to reduce the spread of COVID-19 and to protect vulnerable populations during a public health crisis. Our objective was to examine how three bans on eviction enforcement impacted eviction filings from March 2020 through January 2022 in Ontario, Canada.

**Methods:**

Data were derived from eviction application records kept by the Ontario Landlord and Tenant Board. We used segmented regression analysis to model changes in the average weekly filing rates for evictions due to non-payment of rent (L1 filings) and reasons other than non-payment of rent (L2 filings).

**Results:**

The average number of weekly L1 and L2 applications dropped by 67.5 (95% CI: 55.2, 79.9) and 31.7 (95% CI: 26.7, 36.6) filings per 100,000 rental dwellings, respectively, following the first ban on eviction enforcement (*p* < 0.0001). Notably, they did not fall to zero. Level changes during the second and third bans were insubstantial and slope changes for L2 applications varied throughout the study period. The L1 filing rate appeared to increase towards the end of the study period (slope change: 1.3; 95% CI: 0.1, 2.6; *p* = 0.0387).

**Conclusion:**

Our findings suggest that while the first ban on eviction enforcement appeared to substantially reduce filing rates, subsequent bans were less effective and none of them eliminated eviction filings altogether. Enacting upstream policies that tackle the root causes of displacement would better equip jurisdictions during future public health emergencies.

**Supplementary Information:**

The online version contains supplementary material available at 10.17269/s41997-023-00813-1.

## Résumé

**Objectif:**

Les ordonnances d’expulsion résidentielles ont été suspendues pour réduire la propagation de la COVID-19 et pour protéger les populations vulnérables pendant une crise de santé publique. Notre objectif était d’examiner l’impact de trois interdictions d’exécution d’ordonnance d’éviction sur les requêtes d’expulsion de mars 2020 à janvier 2022 en Ontario, au Canada.

**Méthodes:**

Les données ont été tirées des dossiers de requêtes d’expulsion conservés par la Commission de la location immobilière de l’Ontario. Nous avons utilisé une analyse de régression segmenté pour modéliser les changements dans les taux hebdomadaires moyens de requêtes d’expulsion pour non-paiement du loyer (requêtes L1) et pour des raisons autres que le non-paiement du loyer (requêtes L2).

**Résultats:**

Le nombre moyen de demandes hebdomadaires de dépôts de requêtes L1 et L2 a chuté de 67,5 % (IC à 95% : 55,2, 79,9) et de 31,7 % (IC à 95% : 26,7, 36,6) pour 100 000 logements locatifs, respectivement, suite à la première interdiction d’exécution des expulsions. Il est à noter qu’elles ne sont pas tombées à zéro. Les changements du taux de requêtes au cours de la deuxième et la troisième interdictions n’étaient pas substantiels et les changements de pente pour les applications L2 ont varié tout au long de la période d’étude. Le taux de dépôt de L1 a semblé augmenter vers la fin de la période d’étude (changement de pente : 1,3; IC à 95% : 0,1, 2,6; *p* = 0,0387).

**Conclusion:**

Nos résultats suggèrent qu’alors que la première interdiction d’exécution des expulsions a semblé réduire considérablement les taux de dépôt d’expulsion, les interdictions ultérieures ont été moins efficaces et aucune d’entre elles n’a complètement éliminé les dépôts d’expulsion. L’adoption de politiques en amont qui s’attaquent plutôt aux causes profondes des expulsions permettrait de mieux équiper les juridictions lors de futures urgences de santé publique**.**

## Introduction

The COVID-19 pandemic has exacerbated health and financial inequities created by long-standing social and economic policies. Marginalized communities have experienced disproportionately high rates of COVID-19 morbidity and mortality in addition to pandemic-related wage and job loss, increasing vulnerability to hardships such as food and housing insecurity (Gupta & Aitken, 2020; Karmakar et al., 2021; Mude et al., 2021; Ali et al., 2020; Patrick et al., 2020). One estimate indicates that 5% of Canadian renters were behind on rent during the first year of the pandemic, putting more than 270,000 households at risk of eviction (CMHC, 2023; Tranjan, 2021).

Eviction, which most often refers to the forceable expulsion of a tenant from a landlord’s residence, is a severe outcome of housing insecurity with significant repercussions for public health. Individuals facing eviction or even the threat of eviction are more likely to experience substandard housing or homelessness, psychological distress (e.g., depression, anxiety, suicide), exposure to harmful substances (e.g., lead, asbestos, mold), and violence, as well as medical care and social network disruptions (Desmond, 2012; Grainger, 2021; Desmond, 2015; Desmond & Kimbro, 2015; Fowler et al., 2014; Hoke & Boen, 2021; Jacobs, 2011; Krieger & Higgins, 2002; Marquez et al., 2019; Rojas & Stenberg, 2016; Vásquez-Vera et al., 2017). Physiological and behavioural responses to eviction can compromise immunity and overall health, rendering affected individuals more vulnerable to COVID-19 and other infectious diseases (Benfer et al., 2021; Hatch & Yun, 2021; Vásquez-Vera et al., 2017). Coping strategies—such as doubling up or seeking homeless shelters—increase transmission opportunities among evicted tenants and their communities alike (Benfer et al., 2021).

Many governing bodies acknowledged the important role of housing stability in containing COVID-19 by introducing eviction moratoria at the onset of the pandemic (OECD, 2021). Emerging research suggests that these moratoria were successful at reducing both eviction rates and infections (Leifheit et al., 2021; Nande et al., 2021; OECD, 2021). However, this collection of work does not extend beyond 2020, despite the continuation or re-instatement of numerous bans in subsequent years. Extended evaluations are needed to determine the long-term effects of such legislation, including whether and how its potency varies over time. The goal of this analysis is to examine how three temporary bans on eviction enforcement impacted rates of eviction filings from March 2020 through January 2022 in Ontario, Canada.

## Methods

### Setting

Ontario is home to over 14 million people—nearly 40% of Canada’s total population (Statistics Canada, 2017). Thirty percent of households are renters and 10% are considered low-income (Statistics Canada, 2017, 2022). Over the past decade, rapid economic and population growth, coupled with reduced affordability of homeownership, have dramatically increased the demand for rental housing and consequentially driven up costs throughout the province (Urbanation, 2020). Notably, economic growth has primarily been concentrated among high-income earners. The median wage of Ontario renters has decreased, creating a precarious rental market for lower-income tenants who are disproportionately racialized (Leon & Iveniuk, 2020). Although the growing housing crisis had not manifested in increased overall eviction filings prior to the COVID-19 pandemic (see Supplementary Material, Appendix A), a considerable threat of displacement loomed. The most recent estimates from this period suggest that nearly 50% of renters were paying unaffordable rental housing costs in 2018 (Advocacy Centre for Tenants Ontario, 2018).

### Policy intervention

On March 19, 2020, the Ontario Landlord and Tenant Board (LTB) suspended “all hearings related to eviction applications, unless the matter relate[d] to an urgent issue such as an illegal act or serious impairment of safety” (Tribunals Ontario, 2020). This ban was lifted on September 14, 2020, and all proceedings resumed virtually.

In light of the 2021 winter surge of COVID-19 infections and resulting stay-at-home order, a second ban on eviction enforcement was instated on January 14, 2021. Unlike the first ban, however, it was not uniformly lifted throughout the province. Hastings-Prince Edward, Kingston, Frontenac, and Lennox & Addington, and Renfrew County and District were the first regions to end their bans on February 10, 2021; Toronto, Peel, and North Bay-Parry Sound were the last, on March 8, 2021. Finally, a third ban on eviction enforcement was instated on April 8, 2021, and ended on June 2, 2021. Virtual eviction hearings were permitted during both the second and third bans.

### Data source

Two study team members (A.H., A.P.) used the *Freedom of Information and Protection of Privacy Act* (Information and Privacy Commissioner of Ontario, 2014) to request the records of all eviction applications submitted to the LTB of Tribunals Ontario between January 1, 2017, and January 31, 2022. To accomplish this, they emailed a completed records request form and cover letter outlining details regarding the requested data and data format, as well as a $5 application fee. Tribunals Ontario approved the request in approximately 30 days and electronically transferred the data after the research team paid a $30 processing fee.

The transferred dataset contained the following information: address of tenant, date of filing, filing type (L1, L2, and/or L4), filing notice (N4, N5, N6, N7, N8, N12, and/or N13 form provided), and the preliminary outcome of the filing (which we chose not to use due to uncertainty regarding the ultimate outcome). L1 applications are filed to evict a tenant for non-payment of rent. L2 applications can be, but are not always, used to evict a tenant for reasons other than non-payment of rent (e.g., causing damage to rental units and overcrowding), which are specified within a filing notice. L4 applications can be used to evict a tenant who has not met the conditions of a mediated settlement order following the submission of an L1 or L2 application. Since we were primarily interested in evaluating changes in initial eviction applications, we omitted all L4 applications and any L2 applications that were not filed with the intention to evict. See Supplementary Material, Appendix B for more details.

This research was deemed exempt by the Unity Health Toronto Research Ethics Board.

### Exposure

**Fig. 1:**
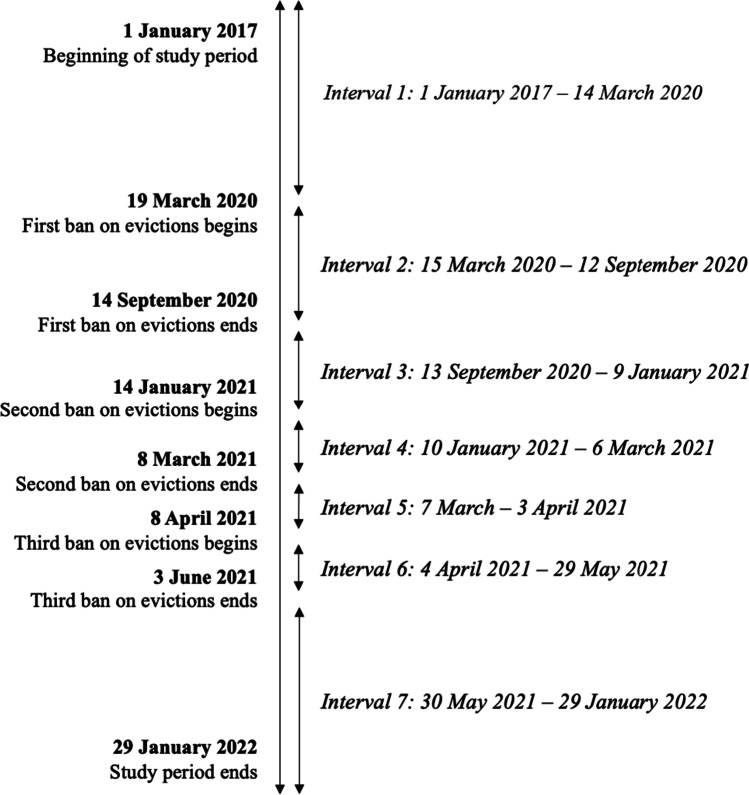
Study timeline

We created seven study intervals to explore the potential impacts of each ban on eviction enforcement. These﻿ intervals encompassed the periods that the three bans—hereby referred to as treatment periods—were in effect, and four control periods that corresponded to the time before, between, and after the bans, estimated in weeks (Fig. 1). This approach allowed us to eliminate variation caused by day-to-day fluctuations but resulted in inexact intervals relative to the dates of each ban.

At the risk of underestimating the potential impacts of the second ban, we classified March 8, 2021, as the final date of the second treatment period to avoid potential spillover effects into the third control period and better power our analyses.

### Outcome

Many pandemic-related eviction bans exclusively applied to non-payment of rent; to maximize the generalizability of our findings, we examined L1 filing trends in isolation. However, we were also interested in how the ban on eviction enforcement influenced L2 filing trends for several reasons: (1) they can serve as “bad faith” alternatives to L1 applications; (2) landlords may have been more stringent about reducing crowding at a time when it also may have been more likely due to lost income; and (3) landlords may have been more likely to observe behaviours they believed warranted eviction due to their, or their tenants’, increased time at home.

We therefore calculated two outcome variables to support our analyses: the average weekly filing rate of L1 and L2 applications per 100,000 rental dwellings. Standardizing the filing rates helped to account for the changing number of rental dwellings throughout the study period.

We first calculated the number of L1 and L2 eviction applications that were filed each week throughout the study period. Next, we approximated the number of rental dwellings that existed during each week of the study period using Canada Mortgage and Housing Corporation’s (CMHC) 2016–2021 Primary and Secondary Rental Market Survey Data (CMHC, 2022a, 2022b). CMHC estimates the annual number of privately owned rental dwellings within jurisdictions of 10,000 people or more during the first 2 weeks of October. Using these estimates as annual benchmarks, we calculated and applied a constant weekly growth rate for each week of the study period. In the absence of the 2022 CMHC data, we used the 2020–2021 weekly growth rate to estimate the number of weekly rental dwellings from late October 2021 through January 2022. Finally, we divided the total number of weekly L1 and L2 applications over the weekly rental dwelling estimates and standardized per 100,000 units.

### Analysis

We used an interrupted time series (ITS) approach to estimate the potential effects of Ontario’s bans on eviction enforcement. ITS is a powerful quasi-experimental method that is well suited for clearly delineated treatment periods and routinely collected, evenly spaced outcomes data.

We first plotted the weekly L1 and L2 eviction filing rates to visually inspect time series patterns throughout the study period. Then, we employed two segmented regressions to gauge changes in the level (magnitude) and slope (trend) of weekly L1 and L2 eviction filing rates for each treatment and control period relative to their counterfactual. In this context, a level change corresponds to the immediate effect of a ban (or lifting of a ban) on filing rates, whereas a slope change corresponds to the sustained effects of the ban (or lifting of a ban) on filing rate trends throughout an interval. Models adjusted for seasonal variation, trends over time (captured by week), and the last week of December, which had considerably lower rates of eviction than any other week throughout the year. We used second-order autoregressive models to account for serial correlation among the weekly observations (Wagner et al., 2002).

Given the brief interlude between the second and third bans, we conducted two secondary analyses that combined intervals 4–6 using the same methodology described above. This approach increased our statistical power, thereby enhancing our ability to detect any meaningful changes in eviction rates throughout the study period.

All analyses were conducted using Stata 15 (Stata Corp, College Station, TX) and SAS (SAS Institute, Cary, NC).

## Results

**Fig. 2:**
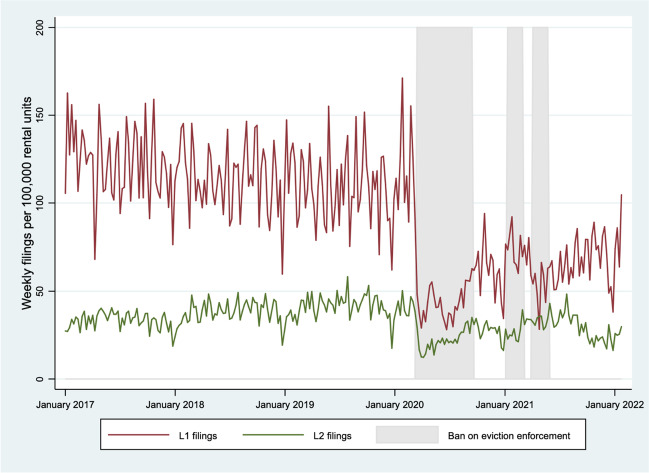
L1 and L2 eviction applications filed in Ontario, Canada, from January 2017 to January 2022

Between January 1, 2017, and January 29, 2022, Ontario landlords filed 211,960 unique L1 and 76,045 unique L2 applications with the intention to evict. While there were substantial fluctuations in both L1 and L2 filings following the bans on eviction enforcement, neither fell below 25 or 10 per 100,000 rental dwellings, respectively, and the filing rate for both appeared to be trending upwards towards the end of the study period (Fig. 2).

### L1 filings

At the onset of the study period, the rate of weekly L1 eviction applications appeared to be steadily declining (slope: − 0.1; 95% CI: − 0.2, − 0.1; *p* < 0.0001) (Table 1). After the first ban on eviction enforcement was implemented, weekly L1 applications fell by 67.6 filings per 100,000 rental dwellings (95% CI: 55.3, 79.9; *p* < 0.0001). In the control period following the first ban on eviction enforcement, weekly L1 applications rose by an average of 23.0 filings per 100,000 rental dwellings (95% CI: 4.9, 41.1; *p* < 0.0001). Though the magnitude and direction of level changes oscillated in subsequent intervals, none of these differences was statistically significant in the main or secondary models (Tables 1 and 2).

**Table 1:** Parameter estimates for average weekly L1 eviction applications filed per 100,000 rental dwellings in Ontario, Canada (main model)

Interval	Time period	Parameter	Estimate (95% CI)	*p*-value
1	1 Jan 2017–14 Mar 2020	Intercept	121.9 (116.3, 127.4)	< 0.0001
		Slope	− 0.1 (− 0.1, − 0.2)	< 0.0001
2	15 Mar 2020–12 Sep 2020	Level change	− 67.6 (− 79.9, − 55.3)	< 0.0001
		Slope change	0.5 (− 0.3, 1.2)	0.2251
3	13 Sep 2020–9 Jan 2021	Level change	23.0 (4.9, 41.1)	0.0136
		Slope change	− 0.8 (− 2.4, 0.8)	0.3257
4	10 Jan 2021–6 Mar 2021	Level change	12.5 (− 15.3, 40.2)	0.3792
		Slope change	− 0.8 (− 5.6, 3.9)	0.7297
5	7 Mar 2021–3 April 2021	Level change	0.3 (− 49.5, 50.0)	0.9909
		Slope change	0.5 (− 15.7, 16.7)	0.9527
6	4 Apr 2021–29 May 2021	Level change	− 13.8 (− 53.3, 25.7)	0.4943
		Slope change	2.8 (− 13.4, 19.0)	0.7351
7	30 May 2021–29 Jan 2022	Level change	− 4.2 (− 26.1, 17.8)	0.7095
		Slope change	− 1.5 (− 6.2, 3.1)	0.5194

Level change = immediate effect of a ban (or lifting of a ban) on filing rates; slope change = sustained effects of the ban (or lifting of a ban) on filing rate trends throughout an interval

Models adjusted for seasonal variation, trends over time, and the last week of December

**Table 2:** Parameter estimates for average weekly L1 eviction applications filed per 100,000 rental dwellings in Ontario, Canada (secondary model)

Interval	Time period	Parameter	Estimate (95% CI)	*p*-value
1	1 Jan 2017–14 Mar 2020	Intercept	128.9 (116.3, 127.4)	< .0001
		Slope	− 0.1 (− 0.1, − 0.2)	< 0.0001
2	15 Mar 2020–12 Sep 2020	Level change	− 67.5 (− 79.9, − 55.2)	< 0.0001
		Slope change	0.5 (− 0.3, 1.2)	0.2258
3	13 Sep 2020–9 Jan 2021	Level change	22.8 (4.7, 41.0)	0.0144
		Slope change	− 0.8 (− 2.3, 0.8)	0.3328
4–6	10 Jan 2021–29 May 2021	Level change	9.2 (− 10.0, 28.4)	0.3478
		Slope change	− 0.4 (− 2.2, 1.3)	0.6433
7	30 May 2021–29 Jan 2022	Level change	5.6 (− 10.2, 21.5)	0.4842
		Slope change	1.3 (0.1, 2.6)	0.0387

Level change = immediate effect of a ban (or lifting of a ban) on filing rates; slope change = sustained effects of the ban (or lifting of a ban) on filing rate trends throughout an interval

Models adjusted for seasonal variation, trends over time, and the last week of December

While we similarly did not observe statistically meaningful slope changes in any interval in the main model (Table 1), we found that the trend of weekly L1 filing rates significantly increased during the final control period in the secondary model (slope change: 1.3; 95% CI: 0.1; 2.6, *p* = 0.0387) (Table 2).

### L2 filings

The only notable level change in L2 eviction applications occurred after the first ban on eviction enforcement (Table 3), in which the average weekly number of applications fell by 31.7 filings per 100,000 rental dwellings (95% CI: 26.7, 36.6; *p* < 0.0001). Yet, filing trends changed dramatically. Unlike L1 applications, the rate of L2 applications increased at the onset of the study period (slope: 0.1; 95% CI: 0.1, 0.1;* p* < 0.0001). It more rapidly increased during the first treatment period (slope change: 0.5, 95% CI: 0.2, 0.8; *p* = 0.0006) and then decreased during the following control period (slope change: − 0.9; 95% CI: − 1.5, − 0.3; *p* = 0.0046) (Table 3). Similar slope changes for intervals 1–3 were observed in the secondary model, along with a significant increase during intervals 4–6 (slope change: 0.7; 95% CI: 0.0, 1.5; *p* = 0.0479) followed by a decrease in the final control period (slope change: − 0.7, 95% CI: − 1.2, 0.2, *p* = 0.0088) (Table 4).

**Table 3:** Parameter estimates for average weekly L2 eviction applications filed per 100,000 rental dwellings in Ontario, Canada (main model)

Interval	Time period	Parameter	Estimate (95% CI)	*p*-value
1	1 Jan 2017–14 Mar 2020	Intercept	28.9 (26.9, 31.0)	< 0.0001
		Slope	0.1 (0.1, 0.2)	< 0.0001
2	15 Mar 2020–12 Sep 2020	Level change	− 31.7 (− 36.6, − 26.7)	< 0.0001
		Slope change	0.5 (0.2, 0.8)	0.0006
3	13 Sep 2020–9 Jan 2021	Level change	5.5 (− 1.5, 12.4)	0.1231
		Slope change	− 0.9 (− 1.5, − 0.3)	0.0046
4	10 Jan 2021–6 Mar 2021	Level change	− 5.1 (14.7, 4.5)	0.2994
		Slope change	1.6 (− 0.1, 3.3)	0.0646
5	7 Mar 2021–3 April 2021	Level change	4.4 (− 9.7, 18.6)	0.5414
		Slope change	− 2.8 (− 7.7, 2.2)	0.2727
6	4 Apr 2021–29 May 2021	Level change	− 2.9 (− 14.7, 8.8)	0.6247
		Slope change	2.2 (− 2.7, 7.2)	0.3797
7	30 May 2021–29 Jan 2022	Level change	− 0.8 (− 8.7, 7.0)	0.8338
		Slope change	− 1.0 (− 2.7, 0.6)	0.2223

Level change = immediate effect of a ban (or lifting of a ban) on filing rates; slope change = sustained effects of the ban (or lifting of a ban) on filing rate trends throughout an interval

Models adjusted for seasonal variation, trends over time, and the last week of December

**Table 4:** Parameter estimates for average weekly L2 eviction applications filed per 100,000 rental dwellings in Ontario, Canada (secondary model)

Interval	Time period	Parameter	Estimate (95% CI)	*p*-value
1	1 Jan 2017–14 Mar 2020	Intercept	29.0 (26.9, 31.1)	< 0.0001
		Slope	0.1 (0.1, 0.2)	< 0.0001
2	15 Mar 2020–12 Sep 2020	Level change	− 31.4 (− 36.4, − 26.3)	< 0.0001
		Slope change	0.5 (0.2, 0.8)	0.0009
3	13 Sep 2020–9 Jan 2021	Level change	5.4 (− 1.7, 12.5)	0.1352
		Slope change	− 0.9 (− 0.1, 2.6)	0.0060
4–6	10 Jan 2021–29 May 2021	Level change	− 1.0 (− 8.4, 6.3)	0.7805
		Slope change	0.7 (0.0, 1.5)	0.0479
7	30 May 2021–29 Jan 2022	Level change	− 1.0 (− 7.2, 5.3)	0.7637
		Slope change	− 0.7 (− 1.2, − 0.2)	0.0088

Level change = immediate effect of a ban (or lifting of a ban) on filing rates; slope change = sustained effects of the ban (or lifting of a ban) on filing rate trends throughout an interval

Models adjusted for seasonal variation, trends over time, and the last week of December

## Discussion

We identified substantial drops in L1 and L2 eviction filings following the first ban on eviction enforcement, although rates never fell to zero. There were no significant decreases during the second and third bans. The muted effects of the latter bans could be the result of several factors, including the resumption of eviction hearings following the first ban, and/or mounting rental arrears amid dwindling financial assistance for tenants. Furthermore, observed increases in L2 filing rates during all three treatment periods and the rise of L1 applications during the final control period may reflect some of the bans’ fundamental constraints. Without addressing the root causes of displacement, temporarily weakening one mechanism for initiating evictions may simply increase the use of another or defer filings until protections have been lifted. Collectively, our findings indicate that Ontario’s three bans on eviction enforcement were initially helpful but ultimately insufficient tools for preventing eviction filings throughout the first two years of the COVID-19 pandemic.

The temporary eviction moratoria were reactive policy measures by definition. Governments responded to an evolving emergency with intersecting implications for health and housing using time-limited measures that appeared to postpone, rather than eradicate, its impacts. The uptick in L1 eviction filings we observed towards the end of the study period may reflect the short-term nature of relief provided by such legislation. Similar trends have also occurred in several jurisdictions throughout the United States, which are now reporting eviction rates that are commensurate with, and in some cases, surpass, their pre-pandemic averages (Hepburn et al., 2020). In Ontario, the expiration of eviction moratoria may have been exacerbated by the enactment of Bill 184, which “streamlined” the L1 eviction process by mandating that landlords attempt to negotiate repayment plans prior to filing for eviction (Ontario.ca, 2020). If a repayment agreement is filed with the Landlord and Tenant Tribunal and conditions are breached, the tenant in question can now be evicted without a hearing. Housing advocates have expressed concerns that this legislation undermines renters’ rights and makes it easier for landlords to evict their tenants (Gibson & Pagliaro 2020). This bill diverges from the understanding that pandemic-related shocks intensified economic need and stand to worsen with increased housing displacement.

Evictions pose numerous mental and physical harms with compounding health and economic impacts, including increased community risk for COVID-19 infections, increased healthcare utilization (and therefore, strain on the healthcare system), loss of productivity, and greater uptake of social services ﻿(Biederman et al., 2022; Desmond & Gershenson, 2016; Himmelstein & Desmond, 2021; Kahlmeter et al., 2018; Sandoval-Olascoaga et al., 2021; Taylor, 2018; Vásquez-Vera et al., 2017). Promoting housing stability is a critical component of pandemic containment and recovery—particularly as COVID-19 and other infectious diseases continue to spread. Pivoting to longer-term tenant protection legislation, as well as broader efforts to mitigate poverty and the limited availability of affordable housing, would provide communities with lasting security and resilience against future public health and economic crises.

This study has several strengths. It is the first population-based assessment of the bans on eviction enforcement in Canada, and longer-term assessment of any ban to our knowledge. Data were derived from a single process that captures all formal filings across the province of Ontario and, therefore, the most comprehensive set of information pertaining to housing displacement in the region. However, our findings should be interpreted in light of several limitations. First, this analysis describes trends in eviction filings, not evictions or rates of displacement. We may have included cases where landlords filed for eviction but did not go through with proceedings, or proceedings that did not result in evictions. However, the filing process still creates a substantial burden for tenants that may lead to involuntary relocation regardless of the outcome (Desmond & Shollenberger, 2015). In addition, while our findings offer lessons that may be applicable in other jurisdictions, they are not fully generalizable due to variations in legislation, housing availability, and housing demand, among other factors. Finally, we made several assumptions to produce weekly rental dwelling estimates, which may have resulted in imprecise standardized rates.

## Conclusion

Our findings suggest that while the first ban on eviction enforcement appeared to substantially reduce filing rates, subsequent bans were less effective and none of them eliminated eviction filings altogether. As we observed, reactive policies may at best have only a temporary effect on housing outcomes, and can unintentionally spur longer-term issues, such as backlogs of potential eviction cases. Future research should explore filing patterns by landlord type (corporate vs. individual), geography, and sociodemographic factors to better understand which groups were most likely to be impacted by evictions throughout the bans and inform targeted long-term planning, as well as resource allocation. Jurisdictions should also strongly consider enacting “upstream” legislation that addresses poverty and the limited availability of affordable housing. Reducing—or potentially even eliminating—the risk of displacement would deeply strengthen community preparedness for future emergencies and bolster public health.

## Contributions to knowledge

What does this study add to existing knowledge?

- Ontario’s initial ban on eviction enforcement appeared to substantially reduce filing rates but was insufficient at eliminating them altogether. The impacts of subsequent bans were insignificant, and the rate of applications filed to evict tenants for non-payment of rent appeared to increase after the final ban was lifted.

What are the key implications for public health interventions, practice, or policy?

- This paper provides evidence that bans on eviction enforcement may have initially helped to reduce evictions but fell short of providing robust tenant protections throughout the first two years of the COVID-19 pandemic. Attending to the root causes of eviction (i.e., poverty and lack of affordable housing) is critical for ensuring sustained housing security and preventing adverse housing-related health outcomes during an ongoing public health emergency.

## Supplementary Information

Below is the link to the electronic supplementary material.

Supplementary file1 (DOCX 18 KB)

Supplementary file2 (DOCX 17 KB)

## Data Availability

The data from which the findings are generated will be made available upon reasonable request to the corresponding author.

## Code availability

N/A.
