# Supplementary material for: Eviction filings during bans on enforcement throughout the COVID-19 pandemic: an interrupted time series analysis
Source: Can J Public Health. 2023 Aug 15;114(5):745–54. doi: 10.17269/s41997-023-00813-1 (PMC10485221; doi:10.17269/s41997-023-00813-1)
Supplement: Supplementary file 1 — Supplementary file1 (DOCX 18 KB) [file 41997_2023_813_MOESM1_ESM.docx]

**Appendix A.**  L1 and L2 applications filed annually in Ontario, Canada, 2009-2021

|  | **‘09-10** | **‘10-11** | **11-12** | **‘12-13** | **‘13-14** | **‘14-15** | **‘15-16** | **‘16-17** | **‘17-18** | **‘18-19** | **’19-20** | **’20-21** |
| --- | --- | --- | --- | --- | --- | --- | --- | --- | --- | --- | --- | --- |
| **L1 Filings** | 54,109 | 53,182 | 54,847 | 54,777 | 52,832 | 49,991 | 48,940 | 49,489 | 47,595 | 46,043 | 44,621 | 24,481 |
| **L2 Filings** | 5,628 | 6,158 | 6,867 | 7,102 | 7,132 | 7,983 | 8,876 | 9,987 | 11,404 | 13,945 | 15,732 | 11,031 |

Data derived from Tribunals Ontario 2009-10 through 2020-2021 Annual Report
