# Supplementary material for: Eviction filings during bans on enforcement throughout the COVID-19 pandemic: an interrupted time series analysis
Source: Can J Public Health. 2023 Aug 15;114(5):745–54. doi: 10.17269/s41997-023-00813-1 (PMC10485221; doi:10.17269/s41997-023-00813-1)
Supplement: Supplementary file 2 — Supplementary file2 (DOCX 17 KB) [file 41997_2023_813_MOESM2_ESM.docx]

**Appendix B**. Description of L1, L2, and L4 applications for eviction in Ontario, Canada

**L1 applications** can be used to evict a tenant for non-payment of rent and to collect rent owed by the tenant. These can only be filed after landlords provide tenants with the following *Notice to End your Tenancy* (N form):

- N4: Notice to End Your Tenancy for Non-payment of Rent

**L2 applications** can be used to evict a tenant or collect money for reasons other than non-payment of rent; not all L2 applications intend to end tenancy. Those that do require landlords to file after providing tenants with at least one of the following *N forms*:

- N5: Notice to End your Tenancy for Interfering with Others, Damage or Overcrowding
- N6: Notice to End your Tenancy for Illegal Acts or Misrepresenting Income in a Rent-Geared-to-Income Rental Unit
- N7: Notice to End your Tenancy for Causing Serious Problems in the Rental Unit or Residential Complex
- N8: Notice to End your Tenancy at the End of the Term
- N12: Notice to End your Tenancy Because The Landlord, a Purchaser or a Family Member Requires the Rental Unit
- N13: Notice to End your Tenancy Because the Landlord Wants to Demolish the Rental Unit, Repair it or Convert it to Another Use

**L4 applications** can be used to evict a tenant and collect money owed for rent or damages 30 days after they have failed to meet the conditions of a mediated settlement or order based on an L1 or L2 form.
